# Nystagmus Parameters of Supine Roll Test Correlates With Prognosis After Repositioning Maneuver in Horizontal Semicircular Canal Benign Paroxysmal Positional Vertigo

**DOI:** 10.3389/fneur.2021.790430

**Published:** 2021-12-06

**Authors:** Jia Yu, Yongzhe Gu, Guilin Meng, Xiaosa Zhu, Wenxie Wang, Xueyuan Liu, Aiping Jin

**Affiliations:** Department of Neurology, Shanghai Tenth People's Hospital, Tongji University School of Medicine, Shanghai, China

**Keywords:** benign paroxysmal positioning vertigo (BPPV), horizontal semicircular canal, supine roll test (SRT), nystagmus parameters, computer-controlled canalith repositioning procedure (CCRP), prognostic factors

## Abstract

**Background:** Positional nystagmus induced by supine roll test is characteristic for diagnosing horizontal semicircular canal benign paroxysmal positional vertigo (HC-BPPV). In this study, we aimed to explore the value of nystagmus parameters in by supine roll test (SRT) as prognostic factors in HC-BPPV.

**Methods:** We retrospectively analyzed the nystagmus parameters of 813 patients diagnosed with HC-BPPV by the SRT model in the SRM-IV system through video nystagmography. Then we used the computer-controlled canalith repositioning procedure (CCRP) mode for treatment. Based on the outcomes, patients were divided into either the cured group or the resistant group. The 1:1 propensity score matching (PSM) was applied to minimize potential selection bias. Then univariable and multivariable analyses were performed to identify the association of nystagmus parameters and the efficacy of CCRP.

**Results:** Among the 813 patients, 99 (12.2%) were classified in the resistant group. The right side of HC-BPPV patients was twice the number of the left side patients (537 vs. 276). PSM is used to pair resistant patients to the cured patients, in which 99 pairs were successfully matched. Results of univariate and multivariate analyses showed that patients in the resistant group have longer latency in the affected side [odds ratio (OR) = 1.231 (1.110–1.366); *P* < 0.001] and slower slow phase velocity (SPV) in the healthy side [OR = 0.957 (0.917–0.999); *P* = 0.045].

**Conclusion:** Nystagmus parameters may represent the characteristics of canalith. HC-BPPV patients with a longer latency in the affected side and slower SPV on the healthy side during SRT have a higher risk of HC-BPPV persisting after a single CCRP.

## Introduction

Benign paroxysmal positional vertigo is a peripheral vestibular disease induced by specific head-position changes and is characterized by recurrent transient vertigo and positional nystagmus (1). Benign paroxysmal positional vertigo (BPPV) is the most common cause of vertigo caused by peripheral vestibular dysfunction (2). An evidence-based study from Taiwan showed that the annual prevalence rate of BPPV was 446.4/100,000 (3). Another study by von Brevern showed that the lifetime prevalence of BPPV was 2.4% (2). The three major subtypes of vertigo are posterior semicircular canal benign paroxysmal positional vertigo (PC-BPPV), horizontal semicircular canal benign paroxysmal positional vertigo (HC-BPPV), and cupulolithiasis of the horizontal canal benign paroxysmal positional vertigo (HC-BPPV-cu) (1), with PC-BPPV accounting for 80–90%, and the latter two accounting for only 10–20% (4). Recent studies have shown that there was an overestimation of the prevalence of PC-BPPV, and an underestimation of the anatomical variations of HC-BPPV, implying that HC-BPPV may be more common than previously thought (5–9). However, only a few studies have evaluated the prevalence, diagnosis, and treatment of HC-BPPV. In addition, the quantitative analysis of positional nystagmus is still rare.

The clinical diagnosis of BPPV depends on different positional tests for different semicircular canals, with the supine roll test (SRT) being considered the gold standard for diagnosing HC-BPPV. Canalith repositioning procedure (CRP) is currently the best evidence-based maneuver and the most effective treatment method for BPPV. It contributes to the return of otolith particles detached from the semicircular canal or the crista ampullaris to the utricle following body position changes. Despite the high rates of success associated with CRP, about 20% of patients are resistant to the procedure (10). In most cases, BPPV is spontaneously resolved within a few weeks or months (11). However, since there is a high incidence of BPPV in the population and it increases the risk of cerebrovascular disease and dementia (12, 13), further research is required on how to prevent its occurrence.

Identifying risk factors for BPPV recurrence may help improve treatment efficacy and clinical outcomes, as well as alleviate the pain of BPPV recurrence. A meta-analysis showed that BPPV recurrence is associated with females, hypertension, hyperlipidemia, osteoporosis, and Vitamin D deficiency (14). However, the risk factors for BPPV recurrence are still controversial.

It has been reported that 20.9% of the patients with dizziness have positional nystagmus (15), but it is still unclear if there is any relationship between characteristic nystagmus and BPPV recurrence (16). Presently, computational automatic repositioning is reported to be more effective than manual repositioning (17). This is because automatic repositioning can quantitatively analyze the parameters of nystagmus by video nystagmography, a utility that can be used as a prognostic factor to determine the efficacy of computer-controlled canalith repositioning procedure (CCRP) treatment. In a previous study, we explored the nystagmus parameters of PC-BPPV in the SRM-IV computational reposition system (18) and found positive results. In this study, we used CCRP to mimic the Barbecue maneuver to evaluate the value of positional nystagmus parameters as prognostic factors for predicting treatment outcomes.

## Methods

### Patients

#### Inclusion Criteria

(I) Patients diagnosed with unilateral HC-BPPV from May 2017 to September 2020.
(II) Written informed consent was obtained from each patient.

#### Exclusion Criteria

(I) Bilateral/multiple BPPV.
(II) History of central nervous system diseases (e.g., trauma, tumors, demyelinating diseases, cerebrovascular disease, etc.).
(III) Severe cardiovascular diseases, arrhythmia, psychiatric disorders, or multisystem functional failure.
(IV) History of inner ear diseases.
(V) Inability to record nystagmus parameters due to eye diseases or closed eyes.

This study was approved by the Shanghai Tenth People's Hospital Ethics Committee. All procedures were performed in accordance with the SRM-IV vestibular function diagnosis and treatment system for BPPV (Byron's Medical Science & Technique Inc., Jinan, China) as previously described (16).

#### Diagnosis

**Figure 1:**
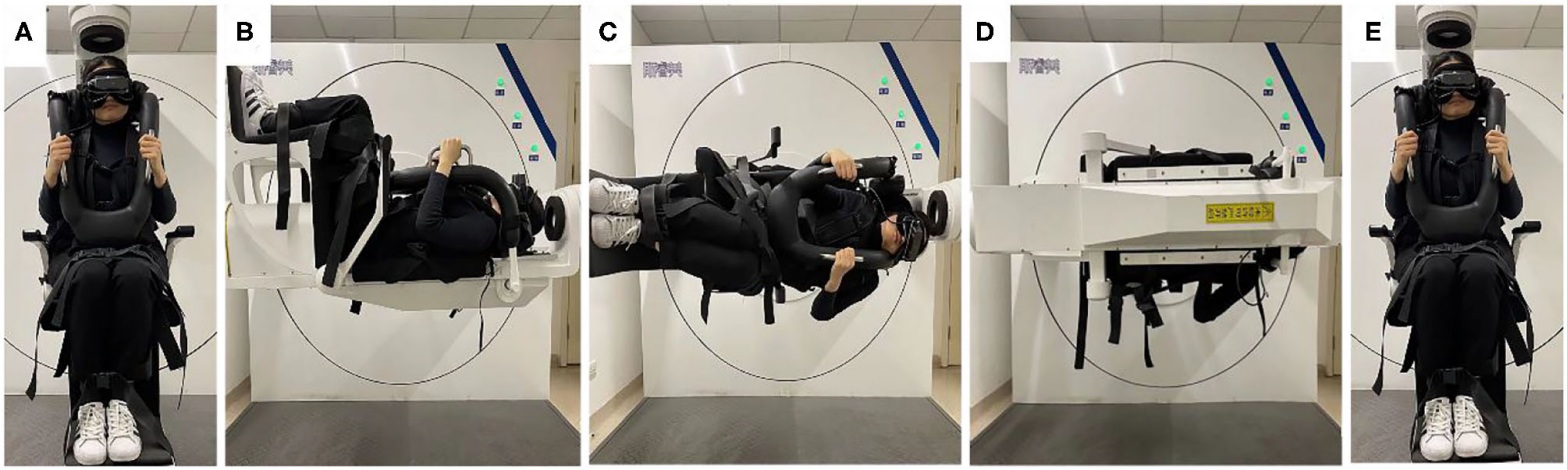
Supine roll test (SRT) mode in SRM-IV system for right horizontal semicircular canal benign paroxysmal positional vertigo (HC-BPPV). In the SRM-IV system SRT mode, each patient was placed in an automatic swivel chair as the starting position **(A)**, shifted to the supine position **(B)**, and then turned left 90° to the left lateral position **(C)**, then turn right 180° to the right lateral position **(D)**. Each action was completed at a speed of 90°/s. Once nystagmus was induced, its parameters were observed and recorded through video nystagmography, including latency, direction, time course, and a max slow phase velocity (SPV) (Figure 2). Then, the patient was slowly returned to the starting position until nystagmus completely disappeared **(E)**.

**Figure 2:**
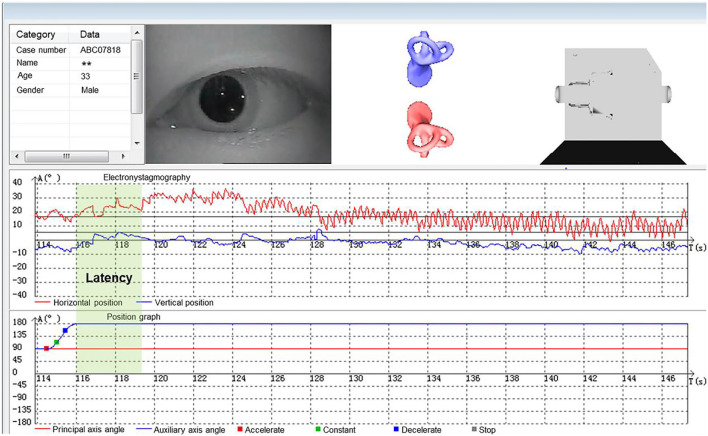
Nystagmus parameters in supine roll test (SRT).

According to the diagnostic criteria established by the Bárány Society in 2015 (1), HC-BPPV was diagnosed using SRT. In the SRM-IV system SRT mode, each patient was placed in an automatic swivel chair as the starting position (Figure 1A), shifted to the supine position (Figure 1B), and then turned left 90° to the left lateral position (Figure 1C), then turn right 180° to the right lateral position (Figure 1D). Each action was completed at a speed of 90°/s. Once nystagmus was induced, its parameters were observed and recorded through video nystagmography, including latency, direction, time course, and a max slow phase velocity (SPV) (Figure 2). Then, the patient was slowly returned to the starting position until nystagmus completely disappeared (Figure 1E). The diagnosis was determined by the direction and duration of nystagmus. Patients were classified as having HC-BPPV when geotropic nystagmus in both directions lasted <1 min, and the nystagmus was more intense with the head turned to the affected side.

#### Treatment

All patients diagnosed with HC-BPPV were treated using the Barbecue maneuver, which is considered a classic method for HC-BPPV reset (19). We used the computer-controlled canalith repositioning procedure (CCRP) mode in the SRM-IV system to mimic the Barbecue maneuver. This helped to avoid the poor cooperation of elderly patients with physical motor impairment or those with cervical spondylosis. For instance, for the right semicircular canal HC-BPPV, in a single CCRP, the patient was first shifted from the supine position to the right-affected side, then rotated 360° to the left 90° at a time at the same speed as SRT mode, held for 60 s at each position, and finally slowly rotated back to the starting position (20). All patients were followed up the next day after CCRP treatment to evaluate the efficacy and repeated SRT to evaluate the efficacy. Patients who had successful treatment after initial CCRP were classified as the “cured group,” while those who did not resolve after initial CCRP were classified as the “resistant group.” Successful treatment refers to the nystagmus and the subjective sensation of vertigo both disappearing. While a positive SRT, the nystagmus or the subjective sensation of vertigo still present, or the nystagmus changed to other types, was considered as resistant BPPV. Those resistant BPPV need specific treatment after corresponding individual CCRP. Subsequent treatment results were not included in the analysis for the efficacy of initial CCRP in this study.

### Statistical Analysis

Student's *t*-test, Mann-Whitney *U*-test, or Chi-squared tests were used for statistical analysis depending on whether the variables were continuous or categorical. Furthermore, the propensity score matching (PSM) was applied to minimize potential selection bias and achieve balanced exposure groups at baseline by taking into account all covariates that may influence the selection of the nystagmus parameters. Propensity scores were estimated using logistic regression using the following covariates: age, gender, blood pressure, affected side, and morbidity seasons. A 1:1 “nearest neighbor” case-control match without replacement was applied, meaning that each resistant patient was matched with one cured patient who had the closest estimated propensity score. Approximate values (within 0.02) were regarded as score-match. Univariate and multivariate analyses were run to identify nystagmus parameters significantly associated with the resistance of benign paroxysmal positional vertigo. All analysis was carried out using SPSS 26 (SPSS Inc., Chicago, IL, USA) and *P* < 0.05 was considered statistically significant.

## Results

**Figure 3:**
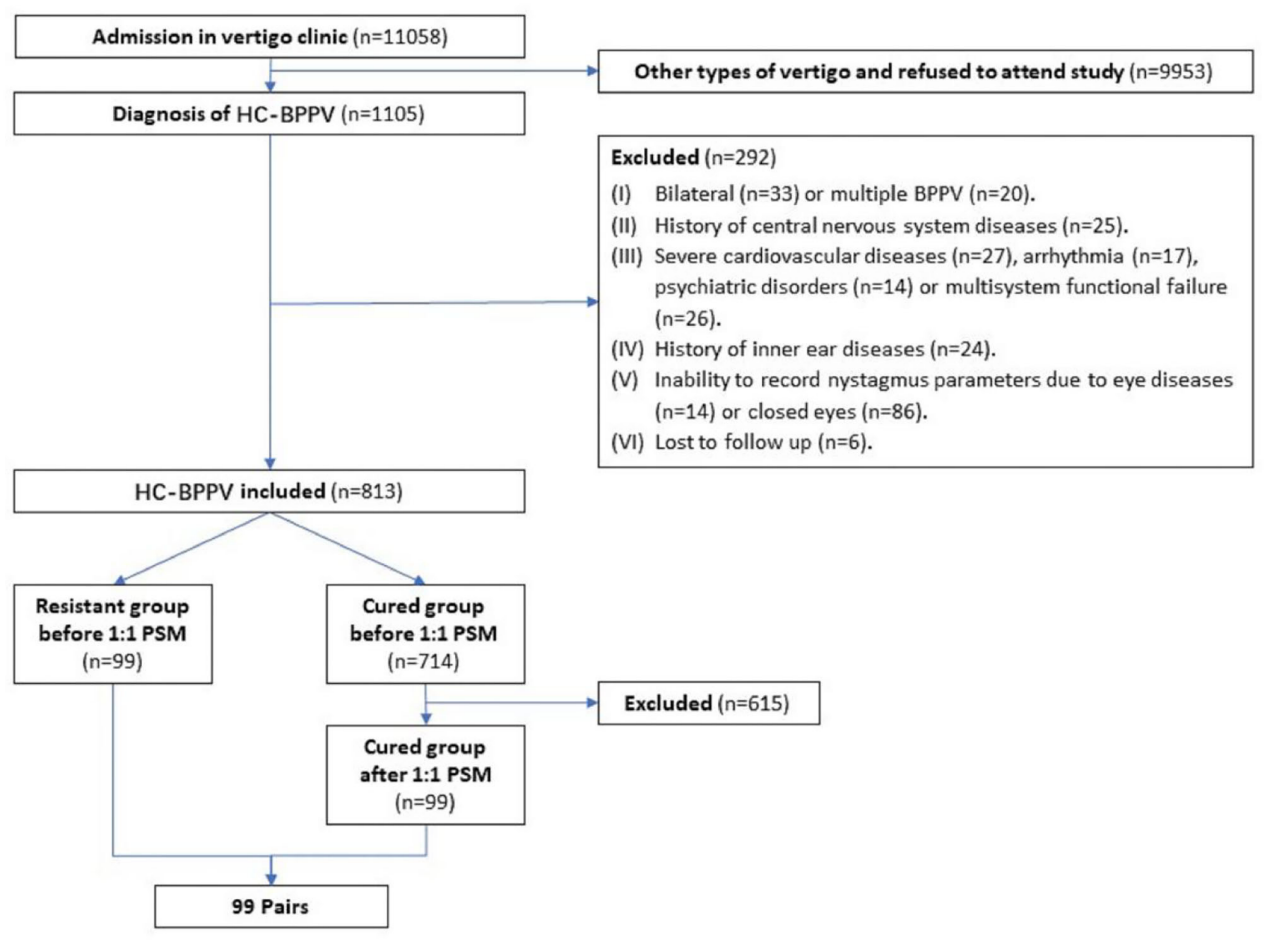
Flow chart of the study implementation. HC-BPPV, horizontal canal benign paroxysmal positional vertigo; PSM, propensity score matching.

Overall, the chief complaint of dizziness of 11,058 patients was related to or aggravated by changes in body position. And all patients, admitted to the vertigo clinic, agreed to be examined in the position test in the SRM-IV system at shanghai tenth people's hospital affiliated with Tongji University from May 2017 to September 2020. Among these, 1,105 patients diagnosed with HC-BPPV were initially interviewed. After following up and recording the nystagmus the next day, a total of 813 patients were finally enrolled, while 292 patients were excluded. Among the 813 patients, 99 (12.2%) were classified in the resistant group, while the remaining 714 (87.8%) were in the cured group (Figure 3). The average age was 56.7 ± 13.4 years old, 255 (31.3%) were male and 277 (34%) were affected on the left side canal.

The baseline characteristics of patients in the resistant group and cured group with and without PSM are shown in Table 1. There were no significant differences in age, sex, blood pressure, and morbidity seasons between both groups. However, there was a difference in the affected canal sides (*P* = 0.01) before PSM, with the right-side HC-BPPV patients being twice more than the left side HC-BPPV patients (537 vs. 276). After matching, 99 cases of the two groups were successfully matched, and each pair was comparable in the above baseline covariates.

**Table 1:** Baseline characteristics of HC-BPPV resistant or cured groups.

**Characteristics**	**Before PSM**			**After PSM**		
	**Resistant group (*n* = 99)**	**Cured group (*n* = 714)**	***P*-value**	**Resistant group (*n* = 99)**	**Cured group (*n* = 99)**	***P*-value**
Age, y, mean (SD)	56.8 ± 11.9	56.7 ± 13.6	0.962	56.8 ± 11.9	57.3 ± 12.9	0.753
**Gender (%)**			0.103			0.518
Male	24 (24.2%)	231 (32.4%)		24 (24.2%)	28 (28.3%)	
Female	75 (75.8%)	483 (67.6%)		75 (75.8%)	71 (71.7%)	
**Blood pressure (mmHg)**						
SBP	132.1 ± 15.2	131.4 ± 16.3	0.696	132.0 ± 15.1	130.4 ± 17.4	0.497
DBP	74.9 ± 9.1	76.6 ± 10.7	0.079	74.8 ± 9.0	74.6 ± 10.1	0.882
**Affected side (%)**			0.010^**^			0.776
Right	54 (54.5%)	483 (67.6%)		54 (54.5%)	52 (52.5%)	
Left	45 (45.5%)	231 (32.4%)		45 (45.5%)	47 (47.5%)	
**Morbidity seasons (%)**			0.639			0.810
Spring	23 (23.2%)	168 (23.5%)		23 (23.2%)	20 (20.2%)	
Summer	31 (31.3%)	195 (27.3%)		31 (31.3%)	28 (28.3%)	
Autumn	19 (19.2%)	176 (24.6%)		19 (19.2%)	24 (24.2%)	
Winter	26 (26.3%)	175 (24.5%)		26 (26.3%)	27 (27.3%)	

*P-values comparing persons in resistant or cured groups. HC-BPPV, horizontal canal benign paroxysmal positional vertigo; PSM, propensity score matching; SD, standard deviation; SBP, systolic blood pressure; DBP, diastolic blood pressure*.

** *P <0.01*.

Table 2 shows a comparison of nystagmus parameters in SRT between HC-BPPV resistant or cured groups after PSM. Compared to the cured group, patients in the resistant group had longer latency in the affected side (3 vs. 0 s, *P* < 0.001) and longer latency on the healthy side (1 vs. 0 s, *P* = 0.001). In addition, those in HC-BPPV resistant group had slower SPV in the affected side (25.6 vs. 33.0°/s, *P* = 0.004) and slower SPV in the healthy side (9.9 vs. 13.4°/s, *P* = 0.005). No significant differences in nystagmus parameters were observed in the supine position. Similarly, no differences were found for bilateral latency difference, SPV difference, or SPV ratio.

**Table 2:** Comparison of nystagmus parameters in SRT between HC-BPPV resistant or cured groups after PSM.

**Characteristics**	**All patients (*n* = 198)**	**Resistant group (*n* = 99)**	**Cured group (*n* = 99)**	***P*-value**
**Supine position**				
Latency	0 (0, 4.0)	0 (0, 4.1)	0 (0, 4.0)	0.773
SPV	8.3 (6.1, 12.5)	7.9 (6.0, 11.7)	9.2 (6.2, 13.5)	0.444
**Affected side**				
Latency	2.0 (0, 5.6)	3.0 (0, 7.0)	0 (0, 2.4)	<0.001^***^
SPV	29.6 (20.9, 40.0)	25.6 (17.5, 36.6)	33.0 (24.0, 41.2)	0.004^**^
**Healthy side**				
Latency	0 (0, 3.0)	1.0 (0, 4.0)	0 (0, 1.6)	0.001^**^
SPV	12.1 (7.8, 18.8)	9.9 (6.6, 16.5)	13.4 (9.2, 19.4)	0.005^**^
Latency difference	0 (−1.0, 2.9)	0.5 (−1.0, 5.0)	0 (−0.9, 1.9)	0.085
SPV difference	15.4 (8.9, 23.2)	13.7 (8.4, 23.2)	17.0 (9.4, 23.9)	0.118
SPV ratio	2.2 (1.7, 3.1)	2.2 (1.8, 3.2)	2.2 (1.6, 3.0)	0.753

*P-values comparing persons in resistant or cured groups. SRT, supine roll test; HC-BPPV, horizontal canal benign paroxysmal positional vertigo; PSM, propensity score matching; SPV, slow phase velocity*.

** *P <0.01*;

*** *P <0.001*.

Univariate and multivariable logistic regression analyses were used to determine the association between nystagmus parameters in SRT and HC-BPPV resistance (Table 3). Multivariate analysis revealed that latency in the affected side and SPV in the healthy side were two independent predictors for resistance in HC-BPPV patients. That is, the longer latency in affected side [odds ratio (OR) = 1.231 (1.110–1.366); *P* < 0.001] and the slower SPV in healthy side [OR = 0.957 (0.917–0.999); *P* = 0.045], the poorer the outcome of the maneuver.

**Table 3:** Univariate and multivariable analyses of nystagmus parameters in SRT between HC-BPPV resistant or cured groups after PSM.

**Characteristics**	**Univariate**		**Multivariate**	
	**OR (95% CI)**	***P*-value**	**OR (95% CI)**	***P*-value**
Latency in supine position	0.996 (0.910–1.089)	0.928		
SPV in supine position	0.980 (0.922–1.042)	0.527		
***Latency in affected side***	1.236 (1.117–1.369)	*** <0.001***	1.231 (1.110–1.366)	<0.001^***^
***SPV in affected side***	0.977 (0.958–0.997)	***0.024***	–	–
***Latency in healthy side***	1.120 (1.030–1.218)	***0.008***	–	–
***SPV in healthy side***	0.956 (0.920–0.993)	***0.021***	0.957 (0.917–0.999)	0.045^*^
Latency difference	1.028 (0.976–1.083)	0.298		
SPV difference	0.983 (0.959–1.008)	0.184		
SPV ratio	1.042 (0.847–1.281)	0.697		

*P-values comparing persons in resistant or cured groups. SRT, supine roll test; HC-BPPV, horizontal canal benign paroxysmal positional vertigo; PSM, propensity score matching; SPV, slow phase velocity*.

* *P <0.05*;

*** *P <0.001. The bold and italics values indicate statistically significant difference*.

## Discussion

### HC-BPPV Recurrence and Risk Factors

Our study shows HC-BPPV recurrence is not correlated with age, gender, seasons, and blood pressure status, which is consistent with some previous studies (21–23), while other studies presented conflicting results, including our previous study on PC-BPPV (16). The results also showed that the recurrence of BPPV was closely associated with the female gender. This is attributed to the lower estrogen levels in older female patients which may decrease the natural bone mass production, resulting in the canalith being unstably connected with the bone labyrinth making it easier to drop and fall (21). The recurrence of HC-BPPV was not associated with gender, leading us to speculate that the horizontal canal might be less affected by estrogen levels.

### HC-BPPV Recurrence and Lateral Side

We found that the right side of HC-BPPV patients was twice the number of the left side of HC-BPPV patients (537 vs. 276), which is in line with the previous studies (24). Lopez-Escámez believed that this might be associated with individual sleeping habits. More people prefer to sleep in the right position which is associated with the affected semicircular canal (25). von Brevern indicated that particles dropped into the semicircular canal depending on the head position (26). On the other hand, we found that right semicircular canal HC-BPPV patients are more likely to be resistant. We speculate that this is due to the effect of gravity. The lateral sleeping position promotes the displacement of canalith particles on the horizontal semicircular canal to one side. It is possible that the canalith particles falling to the right side are more or larger, hence are not easily degraded and are more likely to relapse.

### HC-BPPV Recurrence and Nystagmus Parameters

Nystagmus is closely associated with the vestibular-ocular reflex (VOR). VOR is one of the output reflexes of the vestibular system. The function of VOR is to maintain clear vision through eye movement and connect the extraocular muscles to pull the semicircular canal (27). According to the canalolithiasis hypothesis (28, 29), the movement of free canalith particles in the semicircular canal causes the stimulation or inhibition of vestibular hair cells, and the cupula turned to corresponding orientations due to the different directions of canalith displacement. The abnormal movement of the canalith caused nystagmus through VOR. Therefore, previous studies have proposed that canalith size, movement path, and particle interactions may affect VOR, and are closely associated with nystagmus parameters (30–32). In other words, nystagmus parameters may represent the characteristics of canalith and have value in predicting BPPV recurrence. Previously, there have been qualitative comparisons of the associations between nystagmus and prognosis of HC-BPPV, while quantitative analysis of parameters is rare and controversial. Choi SY suggested that during SRT the appearance of spontaneous reversal of positional nystagmus is independent of the curative effect of restoration in BPPV (33). Lee et al. demonstrated during the lean test that the presence of pseudo-spontaneous nystagmus was uncorrelated to the treatment outcome (31). On the other hand, Kim et al. showed that nystagmus characteristics during the CRP are predictive parameters for the success of the maneuver (9). Our study shows HC-BPPV patients are induced with typical nystagmus in SRT, and the long latency in the affected side and the slower SPV in the healthy side are independently associated with the recurrence of HC-BPPV.

After changing the head position, the canalith particles are affected by gravity and sedimentation, and after exceeding the stimulation threshold of sensory epithelial cells, they move to the lowest point in the semicircular canal, causing nystagmus. The time course from changing the head position to reaching the stimulation threshold to cause nystagmus is called the latency of nystagmus, which is usually between 1 and 5 s (27). In a morphologically descriptive 3- canal biomechanical model, which estimates dynamic cupular and endolymph displacements elicited during HC-BPPV SRT and CRP, the activation latencies in response to SRT were predicted to vary depending upon the initial location of the canalith debris. During SRT, if the particles are initially located in the ampulla rather than in the canal lumen, due to the more time required for the particles to reach the narrow canal lumen, this latency increases. Rajguru et al. applied the model to predict latency changes as a function of particle dimensions and numbers. The particle terminal free-fall velocity at a low Reynolds number varies in proportion to the square of the radius. In this case, single small particles give rise to longer latencies but a much smaller magnitude of response than a single large particle. Our study shows that patients in the resistant group have longer latency in the affected side, which may be associated with the farther away from the canal lumen and the smaller size of the canalith particles (34, 35).

After the latency and the resultant rapid eye movement, the eyeball moves slowly in the opposite direction, which is called the slow phase and is one of the most important physiological characteristics of nystagmus. In a mathematical BPPV model, a large quantity of small canalith particles caused more severe nystagmus than fewer bigger particles (31), so we supposed the resistant group may have a larger number of canalith particles. Our study shows that patients in the resistant group have slower SPV on the healthy side, which may be associated with the small quantity of the canalith particles. And it may be associated with the presence of anatomical variations in the canal diameter and length. In addition, we speculated that in the resistant group the collision of the particles with endolymphatic fluid causes the average velocity to be slower. Moreover, larger studies with longer follow-up and other types of BPPV should be carried out in the future.

## Conclusion

Nystagmus parameters in SRT are associated with resistance to CCRP. This association corresponded to the observation that HC-BPPV patients with a longer latency in the affected side and slower SPV on the healthy side during recording of nystagmus have a higher risk of their BPPV persisting after a single CCRP. It is hoped that the above research results can be helpful for doctors to make clinical diagnoses and judge the prognosis of HC-BPPV patients. Next, our team will conduct a long-term follow-up study on BPPV patients, so as to further practice and promote the current research results.

## Data Availability Statement

The original contributions presented in the study are included in the article/supplementary material, further inquiries can be directed to the corresponding author/s.

## Ethics Statement

The studies involving human participants were reviewed and approved by Committee of Shanghai Tenth People's Hospital approved the study (Ethical Approval Number: SHSY-IEC-4.0/18-44/01). The patients/participants provided their written informed consent to participate in this study. Written informed consent was obtained from the individual(s) for the publication of any potentially identifiable images or data included in this article.

## Author Contributions

JY and XL: conception and design. AJ and XL: administrative support. GM, XZ, and WW: provision of study materials or patients. JY and YG: collection, assembly of data, data analysis, and interpretation. All authors: manuscript writing and final approval of manuscript.

## Funding

This work was supported by the Shanghai Science and Technology Commission (Nos. 20142202900 and 18411961700).

## Conflict of Interest

The authors declare that the research was conducted in the absence of any commercial or financial relationships that could be construed as a potential conflict of interest.

## Publisher's Note

All claims expressed in this article are solely those of the authors and do not necessarily represent those of their affiliated organizations, or those of the publisher, the editors and the reviewers. Any product that may be evaluated in this article, or claim that may be made by its manufacturer, is not guaranteed or endorsed by the publisher.
